# Cell-autonomous and non-autonomous functions of S100A4 in regulating stemness, mesenchymal transition, and metastasis

**DOI:** 10.18632/oncoscience.377

**Published:** 2017-11-28

**Authors:** Bikesh Nirala, David Baskin, Kyuson Yun

**Affiliations:** Department of Neurosurgery, Kenneth Peak Center for Brain and Pituitary Tumors, Houston Methodist Research Institute, Weill Cornell Medical College, Houston, TX 77030, USA

**Keywords:** cancer stem cells, glioblastoma, S100A4, EMT, mesenchymal transition

Glioblastoma (GBM) is the most aggressive and prevalent form of malignant brain cancer and is almost universally incurable. Of the three molecular subtypes of GBM (classical, proneural and mesenchymal (MES)), the MES subtype is the most aggressive and resistant to treatment. Consistently, classical or proneural GBMs often display the MES phenotype at recurrence [1]. Epithelial- mesenchymal-transition (EMT) is associated with therapy resistance and acquisition of stem cell characteristics, including generation of cancer stem cell-like cells. Cancer stem cells (CSCs) are a subset of cancer cells with defining characteristics of normal stem cells that are also endowed with the ability to initiate a tumor upon transplantation. CSCs have been shown to be more resistant to chemo- and radiation therapies compared to bulk tumor cells and generate recurring tumors *in vivo* [2]. Therefore, EMT and CSC describe inter-related therapy-resistant cell states in malignant tumors.

In an earlier study aimed at defining a unique gene signature of glioma stem cells (GSCs), we identified 45 genes whose expression patterns are distinguishable between GSCs and bulk tumor cells and also between GSCs and normal neural stem cells[3]. Among these is *S100a4* (also known as *FSP1/mts-1/metastasin/pEL98*), a gene encoding a small calcium binding protein. S100A4 is expressed in tumor and immune cells, and high S100A4 expression is associated with poor prognosis in virtually all solid tumors. In human cancer tissues, S100A4 protein is observed in the nucleus, cytoplasm or extracellular space where it binds to different binding partners. For example, it binds to p53 in the nucleus to promote p53 degradation and non-muscle MYOSIN IIA in the cytoplasm to promote migration and invasion. Secreted S100A4 binds to ANNEXIN II to promote angiogenesis and RAGE to promote motility and metastasis[4,5].

We recently reported a cell-autonomous function of *S100a4* in promoting self-renewal and MES transition in GSCs[6]. We showed that S100A4+ cells are enriched with long-term self-renewing and tumor-initiating cells in a mouse model of glioma, revealing S100A4 as a novel biomarker of GSCs. In addition, we showed that S100A4 function is critical to maintain self-renewal of human and mouse GSCs, and that selective ablation of GSCs/S100a4+ cells is sufficient to block tumor growth *in vitro* and *in vivo*, supporting the cancer stem cell model. Importantly, we showed that *S100A4* is a master regulator of both EMT and the mesenchymal signature genes in GBM: S100A4 is necessary for the expression of key EMT regulators, *SNAIL2* and *ZEB1*, and *CEBP, FOSL2, RUNX1,bHLHE40*, and *WWTR1/TAZ* – the “master transcriptional regulators” of the MES signature genes. Consistent with these findings, *S100A4* expression is strongly correlated with the MES subgroup and is an independent predictor of MES subgroup GBM patient survival[6]. Interestingly, knocking down S100A4 in human GBM tumorspheres concurrently reduced mesenchymal and increased proneural signature gene expression, suggesting that S100A4 functions as a molecular switch between the two cell states. Molecular mechanism through which S100A4 regulates this process or promotes stemness is currently unknown.

S100A4+ cells in human and mouse GBM are scattered throughout the tumor but often are observed in the perivascular region. The perivascular region is a known niche for GSCs and normal neural stem cells. GSCs and S100A4 have been reported to promote angiogenesis, and S100A4 regulates multiple cytokines shown to promote angiogenesis, including VEGFA and Angiogenin in GSCs (unpublished). Interestingly, others reported that anti-VEGF-resistant gliomas acquire the MES phenotype, increase neutrophil infiltration, and accumulate stem cell-like (Nestin+ Sox2+) cells. Interestingly, infiltrating neutrophils induce S100A4 expression in glioma cells, which is in turn required for MES transition and VEGF- independent angiogenesis in resistant-gliomas[7]. While the acquisition of the MES phenotype with increased S100A4 expression is consistent with our study, we observed that S100A4+ cells in the glioma core do not express normal neural stem cell markers, such as SOX2. These S100A4+ cells are quiescent, and we hypothesize that they are long-term self-renewing GSCs that give rise to short-term self-renewing GSCs, reminiscent of the cellular hierarchy observed in the hematopoietic stem cell system.

Paracrine S100A4 has a critical role in promoting breast cancer metastasis. Genetic deletion of S100A4 in a mouse model of breast cancer showed that S100A4 is necessary for breast cancer metastasis. More recently, two different groups have reported that blocking antibodies against S100A4 can suppress metastasis in a breast cancer model, indicating a paracrine mechanism of S100A4 function in promoting metastasis. One of the reported paracrine mechanisms involves modulation of T-cell infiltration in the primary tumor and the pre-metastatic niche[8]. Whether S100A4 plays a cell-autonomous role in breast cancer metastasis has not been directly tested. It is formally possible that some of the S100A4+/FSP+ cells, presumed to be stromal fibroblasts, are S100A4+ CSCs, which might explain the tumorigenic potential of breast cancer associated fibroblasts.

**Figure 1:**
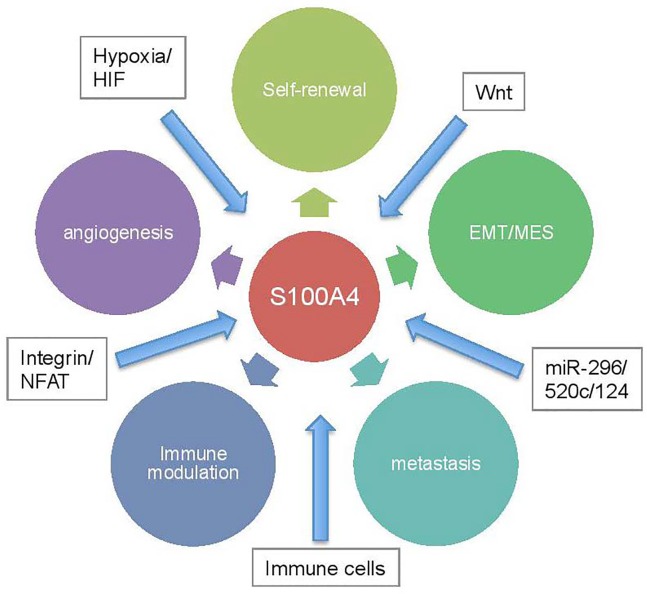
S100A4 expression in malignant cells can be induced by tumor microenvironmental factors, such as hopoxia, immune cells, and Wnt and integrin signaling Its expression can be also modulated by various microRNAs. In turn, S100A4 regulates multiple downstream cellular processes that are critical for tumor progression , such as acquisition of therapy-resistant stem cell state, EMT/mesenchymal transition, metastasis, angiogenesis, and immune cell regulation.

Recent studies reporting the therapeutic potential of S100A4-blocking antibodies suggest that inhibition of paracrine S100A4 function has a great potential for suppressing metastasis. However, inhibiting cell- autonomous functions of S100A4, including promoting stemness and MES transition, will likely require small molecule inhibitors that can be taken up by cancer cells. Such molecules, in combination with standard chemo- and radiation-therapy, may be effective in improving cancer outcome by suppressing MES transition, stemness acquisition, angiogenesis, and metastasis in malignant tumors (Figure 1).
